# Improved cardiovascular risk prediction in patients with end-stage renal disease on hemodialysis using machine learning modeling and circulating microribonucleic acids

**DOI:** 10.7150/thno.46123

**Published:** 2020-07-09

**Authors:** David de Gonzalo-Calvo, Pablo Martínez-Camblor, Christian Bär, Kevin Duarte, Nicolas Girerd, Bengt Fellström, Roland E. Schmieder, Alan G. Jardine, Ziad A. Massy, Hallvard Holdaas, Patrick Rossignol, Faiez Zannad, Thomas Thum

**Affiliations:** 1 Institute of Molecular and Translational Therapeutic Strategies (IMTTS), Hannover Medical School, Hannover, Germany.; 2 Translational Research in Respiratory Medicine, University Hospital Arnau de Vilanova and Santa Maria, IRBLleida, Lleida, Spain.; 3 CIBER of Respiratory Diseases (CIBERES), Institute of Health Carlos III, Madrid, Spain.; 4 Geisel School of Medicine, Dartmouth College, Hanover, NH, USA.; 5 REBIRTH Center for Translational Regenerative Medicine, Hannover Medical School, Hannover, Germany.; 6 Université de Lorraine, Inserm, Centre d'Investigations cliniques-plurithématique 1433, Inserm U1116; CHRU Nancy; F-CRIN INI-CRCT network, Nancy, France.; 7 Department of Medical Sciences, Renal Unit, Uppsala University Hospital, Uppsala, Sweden.; 8 Department of Nephrology and Hypertension, University Hospital, Friedrich-Alexander-University Erlangen-Nürnberg (FAU), Germany.; 9 Institute of Cardiovascular and Medical Sciences, University of Glasgow, Glasgow, United Kingdom.; 10 Division of Nephrology, Ambroise Paré University Medical Center, APHP, Boulogne Billancourt, F-92100 Paris, France.; 11 INSERM U1018, Team 5, CESP (Centre de Recherche en Épidémiologie et Santé des Populations), Paris-Saclay University, Paris-Sud University and Paris Ouest-Versailles-Saint-Quentin-en-Yvelines University (UVSQ), F-94800 Villejuif, France.; 12 Department of Transplantation Medicine, Rikshospitalet, Oslo University Hospital, Oslo, Norway.

**Keywords:** Biomarker, Cardiovascular risk, Hemodialysis, Kidney disease, Machine learning, microRNA.

## Abstract

**Rationale:** To test whether novel biomarkers, such as microribonucleic acids (miRNAs), and nonstandard predictive models, such as decision tree learning, provide useful information for medical decision-making in patients on hemodialysis (HD).

**Methods:** Samples from patients with end-stage renal disease receiving HD included in the AURORA trial were investigated (n=810). The study included two independent phases: phase I (matched cases and controls, n=410) and phase II (unmatched cases and controls, n=400). The composite endpoint was cardiovascular death, nonfatal myocardial infarction or nonfatal stroke. miRNA quantification was performed using miRNA sequencing and RT-qPCR. The CART algorithm was used to construct regression tree models. A bagging-based procedure was used for validation.

**Results:** In phase I, miRNA sequencing in a subset of samples (n=20) revealed miR-632 as a candidate (fold change=2.9). miR-632 was associated with the endpoint, even after adjusting for confounding factors (HR from 1.43 to 1.53). These findings were not reproduced in phase II. Regression tree models identified eight patient subgroups with specific risk patterns. miR-186-5p and miR-632 entered the tree by redefining two risk groups: patients older than 64 years and with hsCRP<0.827 mg/L and diabetic patients younger than 64 years. miRNAs improved the discrimination accuracy at the beginning of the follow-up (24 months) compared to the models without miRNAs (integrated AUC [iAUC]=0.71).

**Conclusions:** The circulating miRNA profile complements conventional risk factors to identify specific cardiovascular risk patterns among patients receiving maintenance HD.

## Introduction

Cardiovascular disease (CVD) represents a major cause of morbidity and mortality among patients receiving maintenance hemodialysis (HD) 1, 2. Risk assessment in this patient population remains challenging. Patients with end-stage renal disease are often excluded from clinical trials due to their medical complexity 3. Furthermore, the pathophysiology of the disease is highly heterogeneous and the relationship between the pattern of risk factors and outcomes is unique and atypical 4, 5. Contrary to the general population in which conventional risk factors are well-established predictors, non-conventional risk factors seem to play a higher prevalent role in patients receiving HD 6, 7. Accordingly, algorithms developed in the general population cannot be directly adopted due to suboptimal performance in patients on HD 8. Advances directed to improve risk assessment are therefore fundamental.

Circulating microribonucleic acids (miRNAs) have emerged as a powerful approach to assist in medical decision-making 9. Based on their biological characteristics, the circulating miRNA concentration and composition may reflect pathophysiological states and offer insights into the molecular phenotype of the patient 10, 11. miRNA-based tests can be developed in minimally invasive liquid biopsies and constitute a cost-effective tool for risk assessment 12, 13. A number of studies have proposed molecular profiling of circulating miRNAs as a source of prognostic indicators for cardiovascular risk stratification in primary 14 and secondary prevention 15, 16. Despite the enormous potential as biomarkers, available data on miRNAs in HD patients is limited.

Using classification tree models based on the so-called chi-squared automatic interaction detector (CHAID) algorithm, we have recently proposed the use of circulating miRNAs to identify specific subpopulation of patients in the context of coronary heart disease (CHD) 17 and cardiometabolic-related conditions 18. Decision trees have become popular in medical research and decision-making. They are conceptually simple, can be constructed through accessible techniques and provide high classification accuracy. Regression trees provide clear decision rules which allow to split the patients in risk groups. Compared to other machine learning methods such as Support Vector Machine (SVM) or Artificial Neural Networks (ANNs), the information provided in tree diagrams is easy to be read and interpreted. Here, we created regression tree models using the decision tree building algorithm adapted to time-to-event data Classification and Regression Trees (CART) algorithm 19. CART modeling explores the effects of high-order interactions among predictors (categorical or continuous), selects the most relevant predictors to construct the decision tree model and combines variables and cutoff values that best discriminate between study groups. CART algorithm identifies complex interactions between predictors and the outcome and defines specific risk profiles. It also allows the time-dependent outcome to be right-censored. From a clinical point of view, the use of machine learning modeling is consistent with the new strategy that suggests the application of biomarkers in individuals or subgroups of individuals 20.

This study sought to assess whether plasma miRNAs could improve cardiovascular risk prediction in patients with end-stage renal disease receiving HD. To do so, we used two different strategies: 1) the traditional biomarker evaluation considering a direct linear relationship with limited interactions between predictors; and 2) taking into account high-level interactions and more complex relationships among the biomarkers, and among biomarkers and the outcome, through regression tree models.

## Methods

### Study cohort and the design of the AURORA trial

Study design, patient baseline data and outcomes of the trial A Study to Evaluate the Use of Rosuvastatin in Subjects on Regular Hemodialysis: An Assessment of Survival and Cardiovascular Events (AURORA) have been previously described 21-23. Briefly, 2776 patients, aged 50-80 years, with end‐stage renal disease who had been treated with regular HD for a minimum of 3 months were randomly assigned to rosuvastatin 10 mg daily or matching placebo. The mean follow-up time was 3.2 years. A number of risk factors were measured according to standardized protocols with inclusion of a central biochemical laboratory. The study (clinicaltrials.gov identifier number NCT00240331) was conducted in accordance with the ethical principles of the Declaration of Helsinki, the Good Clinical Practice guidelines of the International Conference of Harmonization and local regulatory requirements at all participating centers.

Since the cardiovascular event rate was similar in both treatment arms 23 and the aim was to develop a prognostic model of cardiovascular risk, both treatment groups were combined in current study.

### Endpoints

The composite endpoint was time to major cardiovascular event (MACE) defined as death from cardiovascular causes, non-fatal myocardial infarction or non-fatal stroke. A sudden, unexpected death was attributed to CHD, definite or suspected, if there was inadequate information to ascribe a non-cardiovascular cause. All events were adjudicated by an independent endpoint committee blinded to treatment allocation.

### Study populations

**Figure 1:**
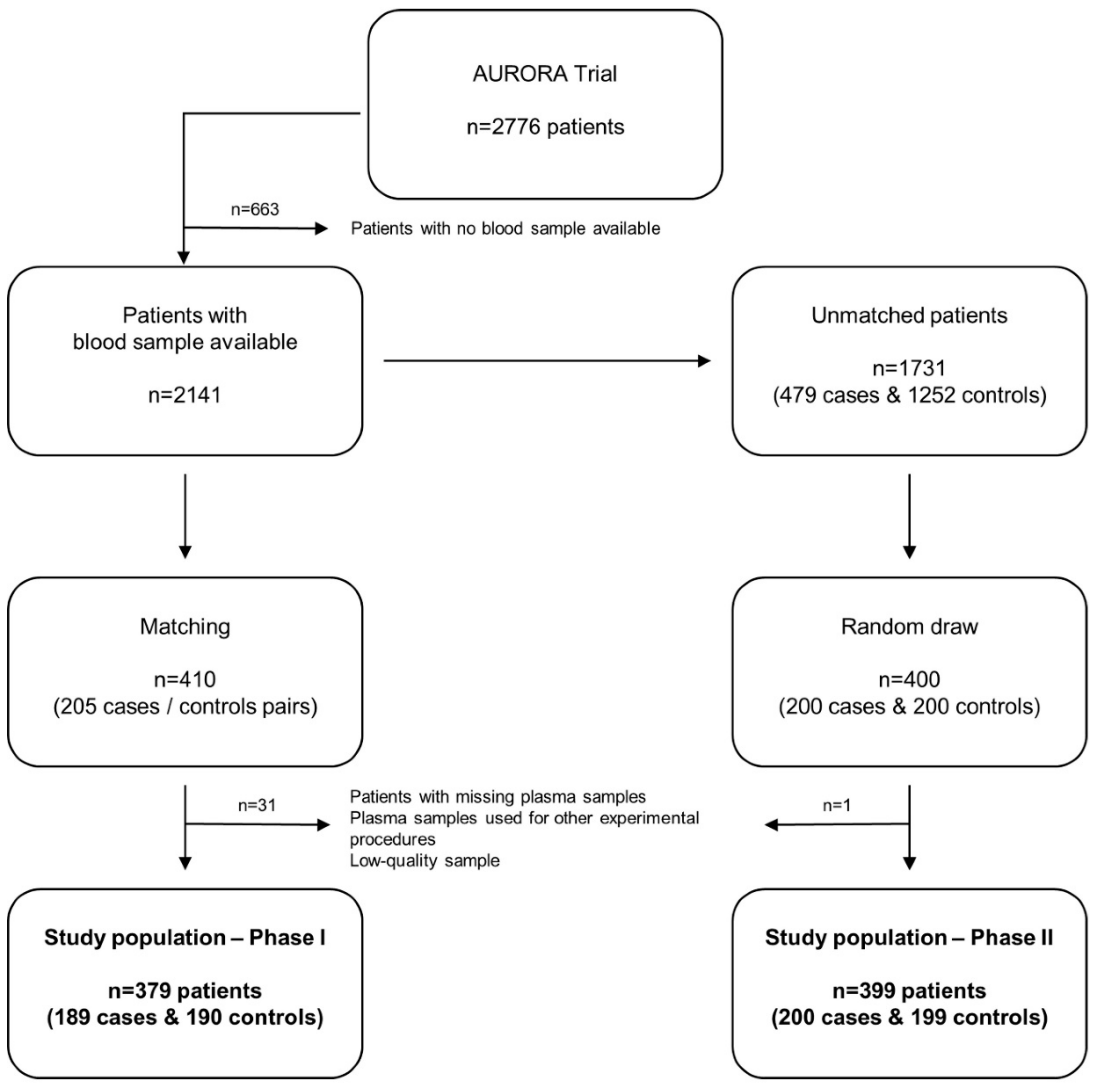
** Study flowchart.** Among the 2776 patients included in the AURORA trial, blood samples were available from 2141 subjects. In Phase I (matching), 205 cases were matched with 205 controls. Patients with missing blood samples, patients whose plasma samples were used for other experimental procedures and patients in which miRNA quantification did not pass the quality control were excluded. The study population in phase I was composed of 379 patients (189 cases, 190 controls). Among the population of unmatched patients with blood sample available (n=1731), 200 cases and 200 controls were randomly drawn (Phase II). One low-quality sample was excluded. The study population in phase II was composed of 399 patients (200 cases, 199 controls).

The study is composed of two clearly defined phases **(Figure 1)**.

#### Phase I

Among the 2776 patients included in the AURORA trial, 205 patients with endpoint (cases) were matched with 205 patients without endpoint (controls) on age, sex, region, body mass index (BMI), KT/V (parameter that quantifies hemodialysis and treatment adequacy. The ratio includes: K = dialyzer urea clearance, t = dialysis time and V = urea distribution volume) and the AURORA Risk Score that includes age, albumin, hsCRP, history of CVD and history of diabetes 24. All patients with missing blood samples (n=8, the full case-control pair was discarded even when there was blood sample from one member of the pair: n=16), patients whose plasma samples were used for other experimental procedures (n=10) and patients in which miRNA quantification did not pass the quality control (n=5) were excluded. The study population in phase I was composed of 379 patients (189 cases, 190 controls). Characteristics of study population are shown in **Supplemental Table S1**.

#### Phase II

Among the population of unmatched patients with blood sample available (n=1731), 200 cases and 200 controls were randomly drawn. One low-quality sample was excluded. The study population in phase II was composed of 399 patients (200 cases, 199 controls). Characteristics of phase II study population and comparison of matching variables and miRNA levels between the two populations (phase I and phase II) are displayed in **Supplemental Table S2 and S3**, respectively.

### miRNA quantification

Different methodological approaches were used for miRNA screening and validation and are described as follows.

#### HTG

The HTG EdgeSeq miRNA Whole Transcriptome Assay (miRNA WTA) (HTG Molecular, Tucson, AZ, USA) was applied to detect miRNAs differentially expressed in plasma samples from 10 cases and 10 matched controls. The HTG EdgeSeq system combines HTG's proprietary quantitative nuclease protection assay (qNPA) chemistry with a new generation sequencing (NGS) platform to enable the semiquantitative analysis of a panel of targeted genes in a single assay. The assay contained 2102 probes, including 2083 miRNAs, 13 housekeeper genes, five negative process controls and one positive process control. Sequencing was performed on the Illumina MiSeq platform. All procedural steps were performed in the appropriate physical locations in accordance with HTG OP No. 0028, Controlled Lab Areas. All samples were prepared in accordance with OP No. 0036, HTG EdgeSeq Sample Preparation.

#### RT-qPCR

RNA was isolated from 150 µL of plasma per sample with the miRNeasy Serum/Plasma Kit (Qiagen, Hilden, Germany) as described by the manufacturer. Synthetic cel-miR-39-3p (1.6x10^8^ copies/µl) (Qiagen) was added to each sample immediately before starting the isolation procedure as a quality control measure of the RNA isolation. RNA was eluted from the columns using 15 µL of water, and 2.5 µL of diluted RNA was used for complementary DNA (cDNA) synthesis with the Reverse Transcription TaqMan MicroRNA Reverse Transcription Kit (Applied Biosystems®, Darmstadt, Germany) according to the manufacturer's instructions. The reverse transcription reaction was then diluted with water (1:3 ratio), and 2 µL was used for quantitative RT-PCR (RT‐qPCR) with specific TaqMan miRNA assays (Applied Biosystems®). Relative miRNA concentrations were calculated using the 2^-dCq^ method with miR-486-5p as an internal standard (d_Cq_ = Cq[miRNA]-Cq[miR-486-5p]), as previously described 25, 26. Samples with Cq≥35 were censored at the minimum observed level for each miRNA. miRNA expression was normalized to the expression of the internal standard, miR-486-5p. miRNA levels were log‐transformed using the base 10 logarithm.

### Statistical analysis

All analyses were performed using R software (the R foundation for Statistical Computing). The two-tailed significance level was set at p<0.05.

#### microRNA level analysis in phase I and phase II

Continuous variables were described as median (interquartile range) and categorical variables as frequencies (percentages). Comparisons of baseline characteristics and miRNA levels between the two populations (phase I and phase II) and between controls and cases in each phase were carried out using non-parametric Wilcoxon test for continuous variables and Fisher's exact test or chi-square test for categorical variables. Heat map of unsupervised hierarchical clustering and principal component analysis (PCA) were used to determine whether the miRNA profile could differentiate between cases and controls 27. Univariate and adjusted associations between miRNAs and the endpoint were assessed using Cox regression models. Hazard Ratios (HR) are presented with their 95% confidence intervals as HR (95% CI). All miRNAs were studied both as continuous and as binary variables (>1^st^ tertile, >median, >2^nd^ tertile). Adjustment variables included all those used for matching performed in phase I: age, gender, region, BMI, KT/V, albumin, hsCRP, history of CVD and history of diabetes. Missing values were reported in phase II for the following continuous variables: KT/V, BMI, albumin and hsCRP. For this reason, adjusted associations between miRNAs and the endpoint were assessed using three different methods: complete observations (i.e. by excluding all patients with missing values for adjustment variables), imputation by the median value and Multiple Imputation Chained Equation (MICE) 28. For MICE, 100 complete datasets were generated and the results of these 100 complete data analyses were pooled. The discrimination ability of each miRNA was assessed using the area under the ROC curve (AUC). Data are presented as the AUC and 95% CI.

#### Regression tree models

In order to study the potential impact of the miRNAs in surrogate or high-order interaction fashion, we performed regression tree models. Particularly, we applied the CART algorithm 19 model and used a bagging 29 procedure (based on 1,000 iterations) for variable selection and error measurement. These flexible machine learning techniques have the potential to capture complex, nonlinear and interactive selection models 19. The extension to the right-censored response proposed by Segal 30 which replace the usual splitting rules with rules based on the Harrington-Flemming classes of two-sample statistics were used. Incidence rates (IR) of event per 100 patients/year summarized the absolute risk. HR was used for representing relative risks within each final node. Kaplan-Meier (KM) curves illustrated differences among groups in the observed time-to-event outcome. As measures of classification accuracy we considered: 1) the integrate area under the cumulative/dynamic receiver-operating characteristic curve (iAUC) 31 of the ordinal risk represented by a hierarchization of the final nodes; and 2) the incidence rate variation index (IRV) defined by 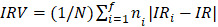, with IR the incidence rate of the population and IR_i_ and n_i_ the incidence rate and the sample size on the i-th final node, respectively (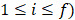, 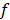 stands for the number of final nodes. The obtained results were employed in an informed stepwise Cox regression model. The predictor importance of the variables considering surrogates effects were computed through the bagging algorithm. R statistical environment (www.r-project.org) was used for these statistical analysis including the survival 32, rpart 33 and nsROC 34 packages.

## Results

### Screening in phase I

**Figure 2:**
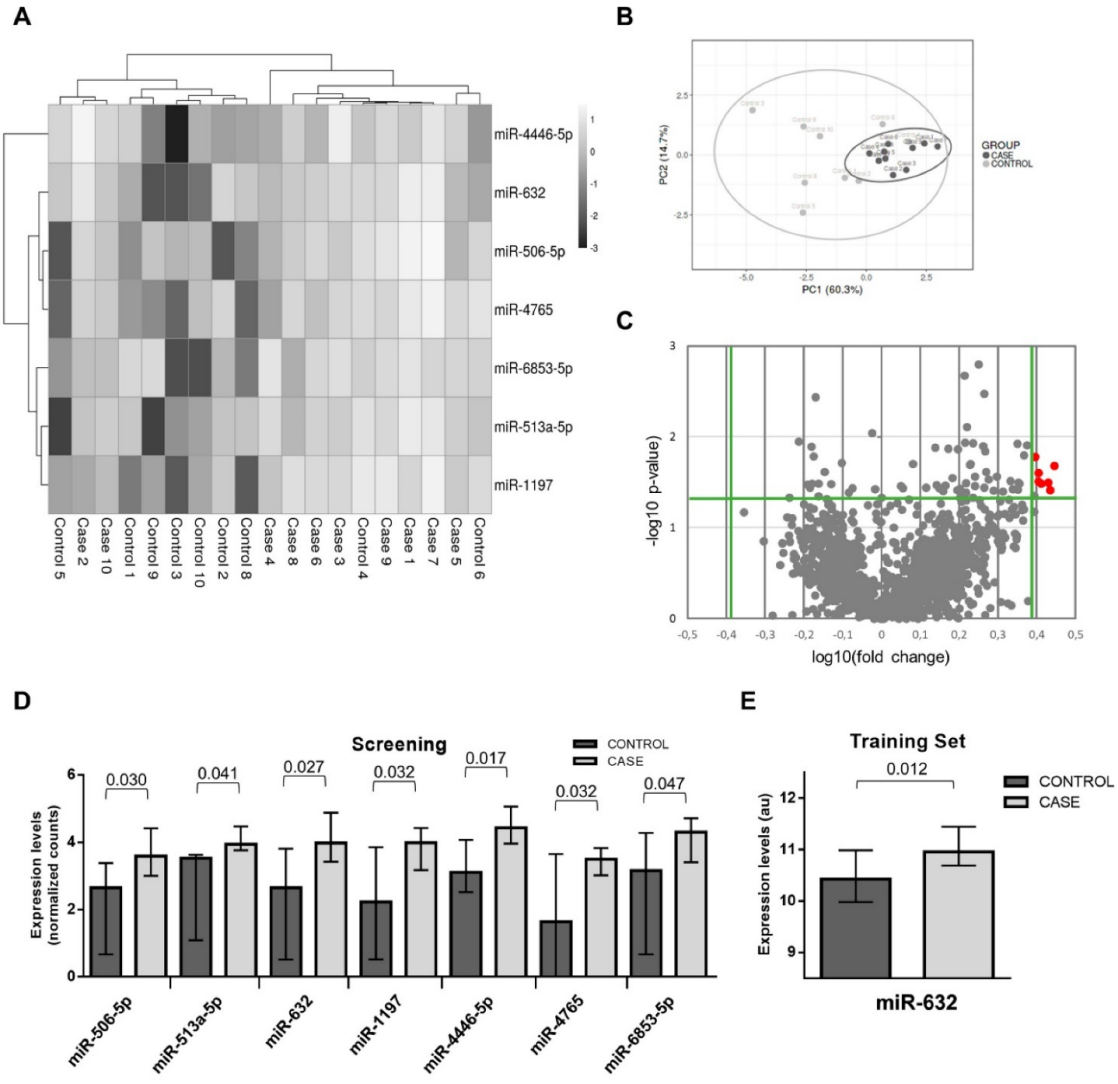
** microRNA screening and selection of microRNA candidates.** A) Heat map showing the unsupervised hierarchical clustering. Each column represents a patient. Each row represents a microRNA; B) Principal component analysis. Each point represents a patient C) Volcano plot of fold change and corresponding p-values for each microRNA after comparison of cases and controls. Each point represent one microRNA. In red microRNA candidates that fulfill the selection criteria; D) Plasma levels of microRNA candidates in the screening study; E) Plasma levels of miR-632 in the training set. Differences between groups were analyzed using non-parametric Wilcoxon test for continuous variables. Data represents the median with interquartile range. P-values describe the significance level for each comparison.

The study flowchart is presented in **Figure 1**. The patient demographics and clinical characteristics for each phase are presented in the **Supplemental Material (Supplemental Table S1-S3)**. miRNA sequencing was performed in randomly selected subgroups of cases and matched controls from phase I using HTG EdgeSeq miRNA WTA Assay technology. One sample (control group) did not pass the quality control and was discarded from further analysis. miRNA candidates were selected according to the following criteria: ≥2.5-fold differential expression and test significance cutoff of p-value less than 0.05. Seven circulating miRNAs met the established criteria: miR-506-5p, miR-513a-5p, miR-632, miR-1197, miR-4446-5p, miR-4765 and miR-6853-5p. As shown in the **Figure 2A-D**, the levels of the seven candidates clearly separated cases and controls.

To corroborate the miRNA sequencing results, we further evaluated the differentially expressed miRNAs by RT-qPCR, the gold standard for circulating miRNA analysis, in a training sample set of 24 cases and 24 matched controls from phase I (including the samples used for miRNA sequencing). We retained miRNAs with a Cq<35 and test significance cutoff of p-value less than 0.05. Six out of seven miRNAs were below the limit of detection in more than 80% of samples (Cq≥35: miR-506-5p, miR-513a-5p, miR-1197, miR-4446-5p, miR-4765 and miR-6853-5p) and were discarded for further analysis. Plasma levels of miR-632 showed stable expression and higher levels in the event group; thus, supporting the sequencing results** (Figure 2E)**.

### Validation in phase I and phase II

Next, we quantified miR-632 in the whole phase I population using RT-qPCR. Due to the low number of miRNA candidates, two additional miRNAs, miR-186-5p and miR-210-3p, known to be associated with cardiovascular risk 35, were also included in subsequent analyses. As shown in **Table 1**, a higher proportion of cases above the median level of miR-632 was observed when compared to patients with no reported event. In addition, miR-632 (>1^st^ tertile and >median) was directly associated with the endpoint, even after adjusting for matching variables **(Table 2)**. Based on these findings, miR-632 was considered as a candidate biomarker for cardiovascular risk in patients on HD.

Next, miR-632 was tested in the phase II cohort (unmatched population). miRNA levels were comparable in cases and controls **(Table 1)**, there were no association between miRNAs and the endpoint in Cox regression models **(Table 3)** and the AUC were low to modest **(Table 4)**. Similarly, no associations were found for miR-186-5p and miR-210-3p, neither in phase I, nor in phase II.

### Regression tree models

Despite the promising findings in phase I with regard to miR-632, results from phase II suggested that circulating miRNAs were not, or were weak, biomarkers for risk stratification in HD patients. We have recently proposed the potential of the miRNA profile to define specific subpopulation of patients in the context of CVD 17, 18. Therefore, we evaluated whether circulating miRNAs could define HD patient subpopulations according to their cardiovascular risk. To this end, we constructed regression tree models using the CART algorithm in the phase II (unmatched) population including the variables that composed the AURORA Risk Score 24: age, albumin, hsCRP, history of CVD and history of diabetes, in addition to the three candidates.

**Figure 3:**
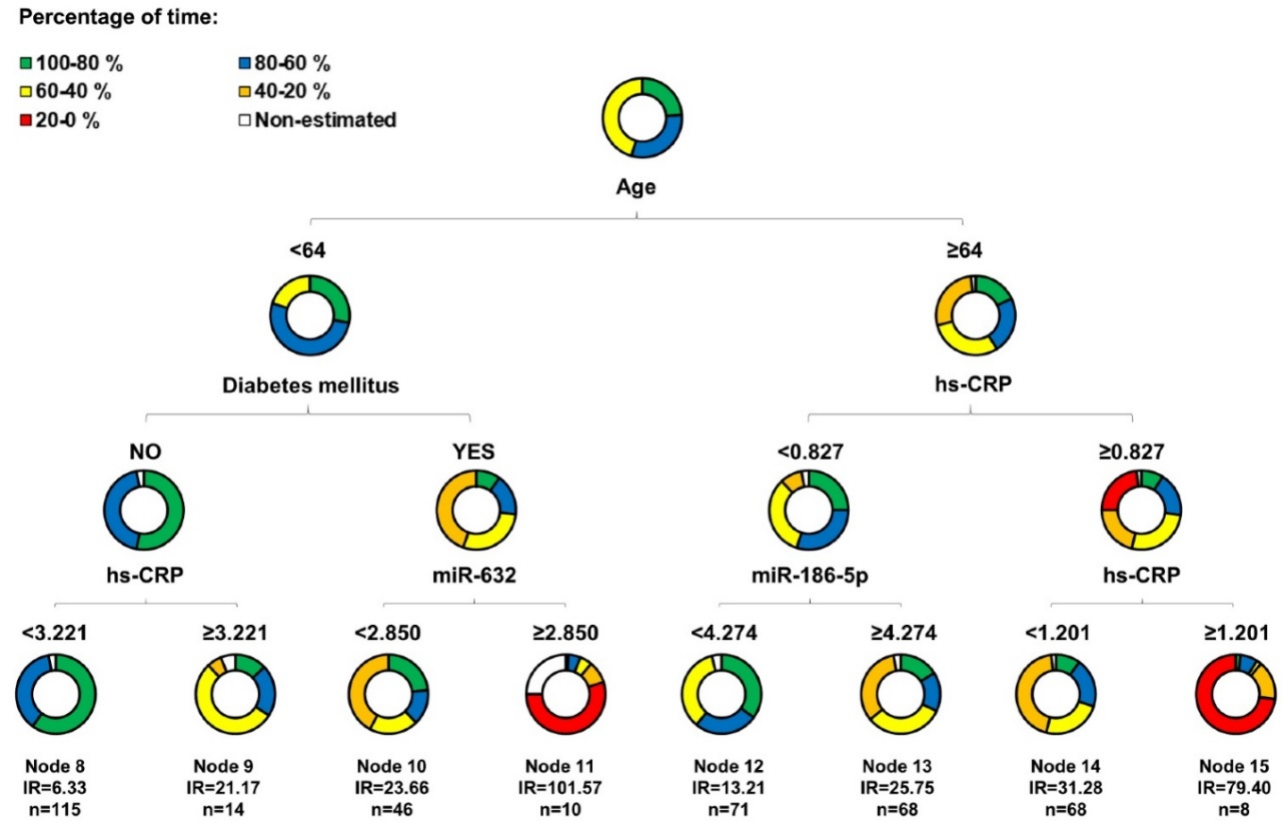
** Decision tree.** Decision tree calculated using the Classification and Regression Trees (CART) algorithm. Predictors considered in the analysis were those that composed the AURORA risk score: age, albumin, hsCRP, history of CVD and history of diabetes, in addition to the three microRNA candidates: miR-186-5p, miR-210-3p and miR-632. The results are presented in a binary decision tree that was constructed by splitting a node into two child nodes repeatedly. Generation of novel nodes was based on the selected predictors and cutoffs. The final nodes are numbered. Incidence rates (IR) of event per 100 patients/year and number of patients per node are included. The length of each color in the bands is proportional to the percentage of the total time that patients are submitted to the risk range. A circle completely green would represent that subjects within this group have a risk of having the event among 0.8 and 1 during the whole follow-up.

**Figure 4:**
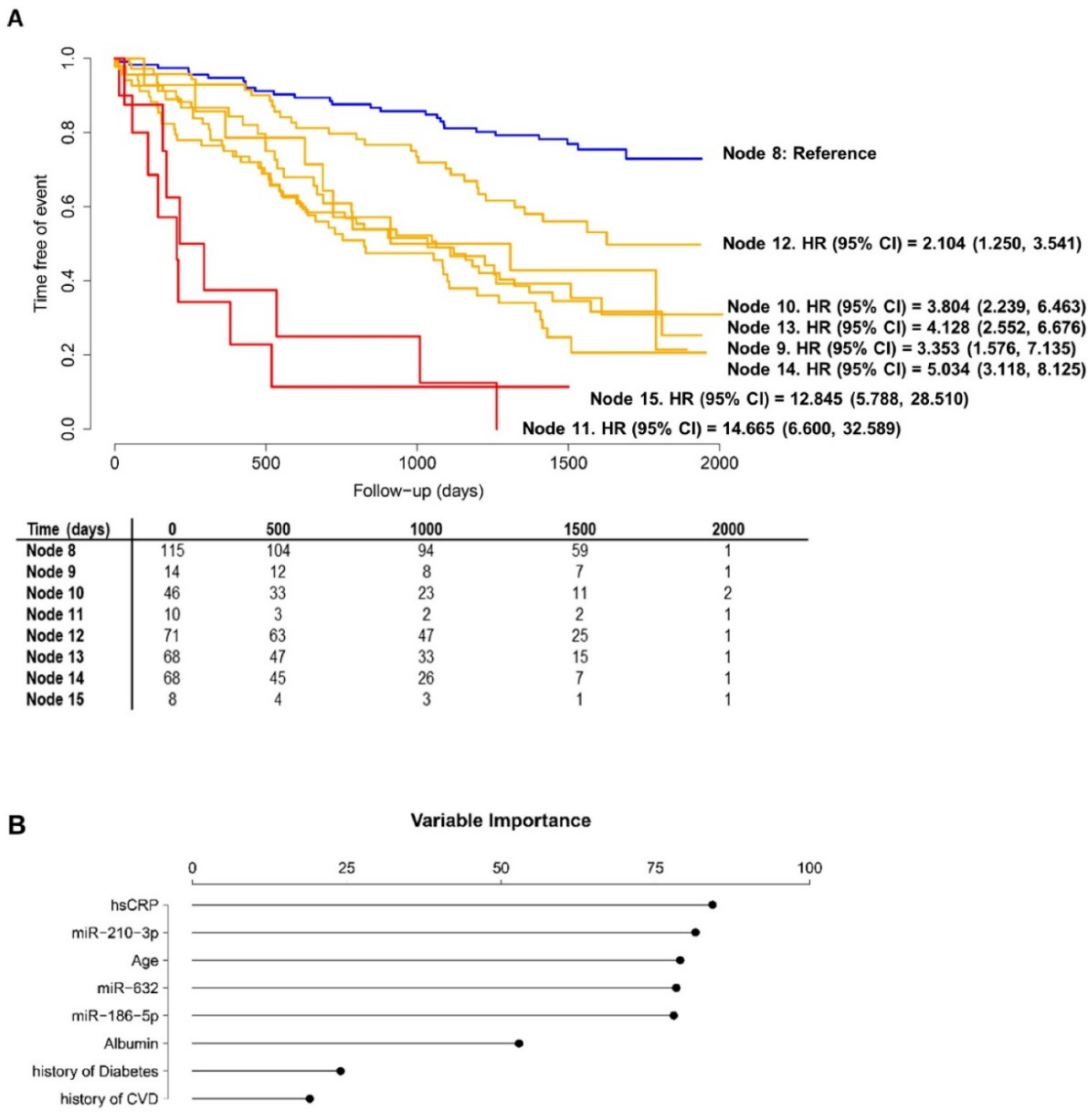
** Performance of the regression tree model.** A) Kaplan-Meier estimations and hazard ratios for the eight final nodes defined by the regression tree model including microRNAs. Data showed as hazard ratios (HR) and their 95% confidence intervals (CI); B) Variable Importance for the variables included in the regression tree model.

**Figure 3** shows the decision tree generated in the phase II population. Circles represent the whole follow-up and the different colors represent the free-event percentage of the patients in the node at that moment. For instance, a totally green circle indicates that 100-80% of the patients within this node do not experience the event during the follow-up. Accordingly, if half of the circle is green and the other half is dark blue, 20% of patients in that node are expected to experience the event during the first half of the follow-up while among an additional 0-20% of them are expected to experience the event during the second half of the follow-up. The percentage of survival at the end of follow-up is above 60%. In node 11, more than 80% of the patients are expected to suffer the event before the third year of follow-up. CART modeling revealed two circulating miRNAs, miR-186-5p and miR-632, that entered at the third level of the regression tree by redefining two risk groups: those patients older than 64 years and with hsCRP<0.827 mg/L and those diabetic patients younger than 64 years. The IR for the final nodes were 6.33, 21.17, 23.66, 101.57, 13.21, 25.75, 31.28 and 79.40. IR values above 100 indicate that all the patients experiment the event before the first year of follow-up. The IRV is 11.63 [8.72-19.24]. Survival Kaplan-Meier curves and hazard ratios from the Cox model for the eight patient subgroups (final nodes) defined by the regression tree model are shown in **Figure 4A**. The iAUC was 0.73 [0.67-0.79], ranging between 0.66 and 0.75 (iAUC for clinical variables without miRNAs = 0.71 [0.66-0.78]). The inclusion of miRNAs allowed a better discrimination during the first two years of the follow-up compared to regression tree models without miRNAs, the difference ranged from iAUC=0.68 for the clinical variables to iAUC = 0.71 for the clinical variables + miRNAs.

The predictor importance of the variables ("variable importance" index) measures the relevance of the variables in a particular model taking into account the potential use as surrogate variables; the role that the variables would play if the main variable was unknown and could not be included in the model. The variable importance analysis revealed the potential of miRNAs as surrogate variables **(Figure 4B)**. In particular, miR-210-3p had an importance similar to hsCRP, although it was not included in the model since hsCRP was already selected. All miRNAs showed a higher importance compared to other components of the AURORA Risk Score such as albumin, history of CVD and history of diabetes.

Since the original objective of the AURORA clinical trial was to analyze the impact of rosuvastatin on clinical outcomes, an additional analysis was performed after inclusion of statin therapy as predictor. Rosuvastatin use was not selected by the regression trees; and therefore, did not interfere with our findings.

## Discussion

Regression tree models suggest the utility of circulating miRNAs in the identification of specific cardiovascular risk profiles among patients with end-stage renal disease receiving maintenance HD.

Initially, we examined miRNAs as biomarkers for risk stratification in HD patients from phase I (matched) and phase II (unmatched). The results from phase I were promising since miR-632 levels were higher in the event group and were independently and directly associated with the endpoint. However, these findings were not confirmed in the phase II validation cohort. In this phase, miR-632 levels were indifferent in both study groups, were not associated with the outcome and, supporting previous data from our group,^17^ showed a low discrimination accuracy (AUC=0.504-0.542). The causes for the discrepancy between the two cohorts remain unclear. Since miRNA quantification was performed in the same laboratory and under highly standardized conditions, the experimental variability between phases may be discarded. One plausible hypothesis could be linked to the characteristics of the study populations. For screening purposes, patients from phase I were strictly matched. For validation, patients from phase II were randomly drawn. Consequently, the clinical features of patients included in phase I and phase II were essentially different. The lack of overlap between the miRNA levels in both phases may reflect the differences related to the clinical status and medication use or even different stages of disease progression. This situation may explain the differences in the association between miRNAs and cardiovascular events and provided the rationale for additional testing. Based on current and previous findings 18, 36, we hypothesized that miRNAs lack biomarker value in the whole population, although such value may exist for selective subpopulations of HD patients. Therefore, we used CART modeling to identify subgroups of patients at different risk of MACE. miR-186-5p and miR-632 entered the regression tree by redefining two risk groups: patients older than 64 years and with hsCRP<0.827 mg/L and diabetic patients younger than 64 years, respectively. Both miRNAs serve as a complement to the demographic and clinical attributes age, hsCRP and history of diabetes in order to determine subgroups of risk that would not be identified without the inclusion of circulating miRNAs.

This study does not only offer additional insights into the potential of circulating miRNAs as biomarkers of cardiovascular risk but also suggest regression tree modeling as an optimal strategy in risk assessment. The advanced statistical technique applied herein revealed eight cardiovascular risk profiles, unknown a priori, based on the predictors considered among our patient population. Regression tree models could be of particular importance in heterogeneous populations, such as HD patients, and conditions in which the conventional risk factors are relevant (e.g. age), but additionally, it could be interesting to define risk subgroups based on other predictors (e.g. miRNAs), such as in cardiovascular risk prediction. By adapting reported indices and plots on the particular problem requirements, regression tree models also emerge as an interesting strategy in the development of biomarkers. The model selected miRNAs as predictors particularly relevant for specific subgroups of individuals whose discriminative power was diluted and of no use for risk assessment when analyzing the whole population.

CVD is the largest contributor to mortality in patients undergoing HD 2. Unfortunately, prediction scores developed for general population or cardiovascular patients, e.g. subjects that have suffered a myocardial infarction, lack accuracy in patients with renal disease 37. Different research groups have identified and proposed specific conventional and non-conventional risk factors 7, 8, 24, 38-43. Nonetheless, these efforts have been mainly focused on established clinical predictors and linear or other parametrically relationships. There is a widespread clinical interest in the development of novel approaches to address the weakness and gaps in the clinical management of patients with renal disease and/or undergoing HD. Here, we describe a simple and precise prognostic model to assess risk stratification in patients with end-stage renal disease receiving HD that integrates a set of readily measurable clinical predictors (age, hsCRP and history of diabetes) and novel biological markers such as miRNAs. The importance of miRNAs for the regression tree models was higher than that observed for other established risk factors such as albumin, history of CVD or history of diabetes 24. miRNAs did not recapitulate the clinical information available, and therefore, may inform risk stratification approaches in those cases in which the clinical history alone does not explain the full complexity of cardiovascular risk. Current findings underscore the potential of using the molecular profiling to guide patient care and optimize the use of healthcare services.

Our results may be of clinical value. Different strategies for patient care, monitoring and treatment can be implemented depending on the risk. A tool to estimate the individual probability of adverse outcome may also be useful for patient selection in clinical trials. In the current study, the eight final nodes of the tree model were composed by subgroups of HD patients with specific clinical and miRNA patterns and marked differences in cardiovascular risks. We suggest that specific disease phenotypes are associated with different risk levels. Thus, cardiovascular risk assessment in patients on HD could be performed in a more personalized approach and should avoid the classical “one disease fits all”. CART modeling created four new subgroups of risk based on miRNAs, which ultimately hold potential to facilitate medical decision-making. We identified one subgroup of very high-risk individuals who may benefit most from adapted intensive monitoring and care (node 11, patients with diabetes, younger than 64 years and miR-632 levels higher than 2.850 arbitrary units: IR=101.57). Conversely, we defined a subgroup of HD patients with low-risk status (node 8, non-diabetic patients younger than 64 years and hsCRP concentration lower that 3.221 mg/L: IR=6.33) in which unnecessary overtreatment should be avoided. Further investigations on the additional benefit of miRNA-based strategies in cardiovascular risk assessment and individualization of decision-making to specific patient subpopulations are warranted.

The fact that miRNAs were associated with cardiovascular events in regression tree models could provide biological insights into disease mechanisms. In the context of atherosclerotic-disease, the cardiomyocyte-enriched miR-186-5p promoted macrophage lipid accumulation and pro-inflammatory cytokine secretion 44 and its circulating levels have been associated with unstable angina pectoris 45. Alterations in plasma miR-632 levels have previously been observed in patients experiencing myocardial infarction 46. miR-210 has been implicated in the mechanisms that regulate the fibrous cap stability of advanced atherosclerotic lesions 47. However, although it cannot be ruled out that miRNAs are risk factors and may be targets for intervention in specific subgroups of patients, these results should be interpreted with caution. The use of statistical models that selected miRNAs in an automated fashion, and in particular, the poor understanding of circulating miRNAs as hormone-like mediators 48, 49, limits causal inferences.

In conclusion, regression tree modeling suggest that the circulating miRNA profile complements conventional predictors in risk stratification and allows the identification of specific cardiovascular risk patterns among patient with end-stage renal disease receiving maintenance HD.

The present study has several strengths. We used a high-quality database based on a large cohort of well-phenotyped HD patients from 280 nephrology centers in 25 different countries. The total sample size was 810 patients, one of the highest sample sizes used in studies focused on the evaluation of non-coding transcriptome as a source of cardiovascular biomarkers, and particularly miRNAs. The retrospective nature of the study constitutes a key limitation. The regression tree model needs to be externally validated in prospective studies. Only 7 of all the 2083 candidates included in the miRNA sequencing were further tested in the training set. From these 7 candidates, only miR-632 was tested in the whole cohort. Other miRNAs may be potential biomarkers of adverse outcomes. The sample size in the screening phase and training set is small. However, it fits with the recommendations for this type of studies 50. In addition, miRNAs are modulators of the statin response 51 and a potential effect of statins on circulating miRNA expression has also been suggested 52. Since the original objective of the AURORA clinical trial was to analyze the impact of rosuvastatin on clinical outcomes, the confounding induced by statin use should not be discarded. Nevertheless, in line with previous findings 17, 18, 53, our additional analysis showed that rosuvastatin use does not affect the association between non-coding RNAs and the outcome. The technical challenges of circulating miRNA quantification should also be taken into account. Currently, there is not a widely accepted normalization system. Based on previous findings 25, 54, we have used miR-486-5p as internal control. We acknowledge that the use of this strategy may be controversial.

## Supplementary Material

Supplementary.

## Figures and Tables

**Table 1:** Plasma microRNA levels in cases and controls from Phases I and II.

	miRNA	Median (Q1-Q3) / n (%)	Median (Q1-Q3) / n (%)	p-value
**Phase I**		**Controls (n=189)**	**Cases (n=190)**	
	miR-186-5p			
	Continuous	4.50 (4.08-4.97)	4.53 (3.97-4.91)	0.57
	> 1^st^ tertile	107 (66.9%)	108 (66.3%)	1.00
	> median	79 (49.4%)	82 (50.3%)	0.91
	> 2^nd^ tertile	54 (33.8%)	53 (32.5%)	0.91
	miR-210-3p			
	Continuous	4.27 (3.97-4.60)	4.31 (4.03-4.77)	0.27
	> 1^st^ tertile	104 (65.0%)	111 (68.1%)	0.56
	> median	76 (47.5%)	85 (52.1%)	0.44
	> 2^nd^ tertile	48 (30.0%)	59 (36.2%)	0.24
	miR-632			
	Continuous	3.98 (3.57-4.47)	4.18 (3.62-4.54)	0.16
	> 1^st^ tertile	115 (62.2%)	132 (71.0%)	0.079
	> median	82 (44.3%)	103 (55.4%)	0.038
	> 2^nd^ tertile	56 (30.3%)	67 (36.0%)	0.27
				
**Phase II**		**Control (n=199)**	**Cases (n=200)**	
	miR-186-5p			
	Continuous	4.44 (3.98-4.79)	4.30 (3.99-4.77)	0.36
	> 1^st^ tertile	133 (68.2%)	128 (65.0%)	0.52
	> median	104 (53.3%)	92 (46.7%)	0.23
	> 2^nd^ tertile	65 (33.3%)	65 (33.0%)	1.00
	miR-210-3p			
	Continuous	4.10 (3.81-4.35)	4.01 (3.72-4.31)	0.20
	> 1^st^ tertile	135 (69.2%)	127 (64.1%)	0.34
	> median	106 (54.4%)	90 (45.5%)	0.087
	> 2^nd^ tertile	65 (33.3%)	66 (33.3%)	1.00
	miR-632			
	Continuous	3.88 (3.41-4.19)	3.83 (3.39-4.14)	0.15
	> 1^st^ tertile	137 (68.8%)	129 (64.5%)	0.40
	> median	103 (51.8%)	96 (48.0%)	0.48
	> 2^nd^ tertile	71 (35.7%)	62 (31.0%)	0.34

p-value from Wilcoxon or Fisher's exact test or chi-square test as appropriate.

**Table 2:** Association between plasma microRNA levels and the endpoint in Phase I.

miRNA	Univariable		Adjusted for matching variables	
	HR (95% CI)	p-value	HR (95% CI)	p-value
miR-186-5p				
Continuous	0.91 (0.74-1.12)	0.38	0.91 (0.73-1.14)	0.42
> 1^st^ tertile	1.00 (0.72-1.38)	1.00	1.02 (0.73-1.41)	0.93
> median	1.03 (0.76-1.40)	0.84	1.07 (0.77-1.48)	0.69
> 2^nd^ tertile	0.94 (0.67-1.30)	0.69	0.96 (0.68-1.34)	0.80
miR-210-3p				
Continuous	1.19 (0.90-1.57)	0.23	1.23 (0.91-1.65)	0.17
> 1^st^ tertile	1.12 (0.80-1.55)	0.51	1.16 (0.83-1.62)	0.39
> median	1.11 (0.81-1.50)	0.52	1.13 (0.82-1.55)	0.45
> 2^nd^ tertile	1.26 (0.92-1.74)	0.15	1.31 (0.94-1.83)	0.11
miR-632				
Continuous	1.17 (0.96-1.43)	0.13	1.19 (0.96-1.46)	0.11
> 1^st^ tertile	1.39 (1.01-1.91)	0.042	1.43 (1.04-1.98)	0.030
> median	1.48 (1.11-1.98)	0.008	1.53 (1.13-2.07)	0.005
> 2^nd^ tertile	1.24 (0.92-1.68)	0.15	1.26 (0.93-1.73)	0.14

HR Hazard ratio; CI Confidence interval.

**Table 3:** Association between plasma microRNA levels and the endpoint in Phase II.

miRNA	Univariable		Adjusted for matching variables (complete observations)		Adjusted for matching variables (with imputation by the median)		Adjusted for matching variables (with MICE)	
	HR (95% CI)	p-value	HR (95% CI)	p-value	HR (95% CI)	p-value	HR (95% CI)	p-value
miR-186-5p								
Continuous	0.90 (0.71-1.14)	0.39	0.95 (0.74-1.21)	0.66	0.89 (0.70-1.13)	0.33	0.89 (0.71-1.13)	0.34
> 1^st^ tertile	0.91 (0.68-1.22)	0.52	0.96 (0.69-1.32)	0.79	0.88 (0.65-1.19)	0.41	0.88 (0.65-1.20)	0.42
> median	0.84 (0.64-1.12)	0.23	0.85 (0.63-1.15)	0.29	0.81 (0.61-1.07)	0.14	0.81 (0.61-1.07)	0.14
> 2^nd^ tertile	1.02 (0.76-1.37)	0.91	1.02 (0.74-1.40)	0.90	0.97 (0.72-1.32)	0.86	0.98 (0.72-1.32)	0.88
miR-210-3p								
Continuous	0.85 (0.65-1.10)	0.22	1.06 (0.79-1.42)	0.68	1.10 (0.83-1.45)	0.49	1.11 (0.84-1.46)	0.47
> 1^st^ tertile	0.84 (0.63-1.12)	0.24	1.06 (0.76-1.47)	0.72	1.06 (0.78-1.45)	0.70	1.07 (0.78-1.46)	0.68
> median	0.74 (0.56-0.98)	0.037	0.84 (0.62-1.15)	0.27	0.87 (0.65-1.17)	0.36	0.87 (0.65-1.17)	0.36
> 2^nd^ tertile	0.98 (0.73-1.32)	0.92	1.06 (0.77-1.47)	0.70	1.10 (0.81-1.49)	0.56	1.09 (0.81-1.48)	0.56
miR-632								
Continuous	0.84 (0.67-1.06)	0.14	0.93 (0.74-1.17)	0.53	0.94 (0.75-1.18)	0.58	0.94 (0.75-1.18)	0.58
> 1^st^ tertile	0.88 (0.66-1.17)	0.38	1.02 (0.74-1.41)	0.90	1.00 (0.74-1.36)	1.00	1.00 (0.74-1.36)	1.00
> median	0.88 (0.67-1.16)	0.37	0.93 (0.68-1.26)	0.62	0.96 (0.72-1.29)	0.81	0.96 (0.72-1.29)	0.80
> 2^nd^ tertile	0.83 (0.61-1.12)	0.22	0.88 (0.64-1.21)	0.43	0.87 (0.64-1.19)	0.39	0.87 (0.64-1.19)	0.38

HR Hazard ratio; CI Confidence interval.

**Table 4:** Discrimination ability of plasma microRNAs in Phase II.

miRNA	AUC (95% CI)	p-value
miR-186-5p		
Continuous	0.517 (0.474-0.560)	0.44
> 1^st^ tertile	0.506 (0.472-0.540)	0.72
> median	0.517 (0.480-0.554)	0.36
> 2^nd^ tertile	0.504 (0.469-0.538)	0.84
miR-210-3p		
Continuous	0.533 (0.490-0.576)	0.14
> 1^st^ tertile	0.522 (0.487-0.557)	0.22
> median	0.540 (0.503-0.576)	0.033
> 2^nd^ tertile	0.501 (0.466-0.536)	0.95
miR-632		
Continuous	0.542 (0.500-0.585)	0.049
> 1^st^ tertile	0.525 (0.489-0.560)	0.17
> median	0.523 (0.487-0.559)	0.21
> 2^nd^ tertile	0.529 (0.496-0.562)	0.085

AUC Area under the ROC curve; CI Confidence interval.

## Abbreviations

AUC: Area under the ROC curve
AURORA: A Study to Evaluate the Use of Rosuvastatin in Subjects on Regular Hemodialysis: An Assessment of Survival and Cardiovascular Events
CART: Classification and regression tree
CHAID: Chi-squared automatic interaction detector
CHD: Coronary heart disease
CVD: Cardiovascular disease
HD: Hemodialysis
iAUC: Integrate area under the ROC curve
IR: Incidence rate
IRV: Incidence rate variation index
hsCRP: High-sensitive C-reactive protein
MACE: Major cardiovascular event
miRNA: microRNA
