# Supplementary material for: Improved cardiovascular risk prediction in patients with end-stage renal disease on hemodialysis using machine learning modeling and circulating microribonucleic acids
Source: Theranostics. 2020 Jul 9;10(19):8665–76. doi: 10.7150/thno.46123 (PMC7392028; doi:10.7150/thno.46123)
Supplement: Supplementary file 1 — Supplementary. [file thnov10p8665s1.pdf]

Supplemental Table S1: Comparison of matching variables between cases and controls in Phase I

	Controls (n=190)	Cases (n=189)	
**Matching variables**	**Median (Q1-Q3) / n (%)**	**Median (Q1-Q3) / n (%)**	**p-value**
Age (years)	67 (60-74)	67 (60-74)	0.94
Sex			1.00
Male	150 (78.9%)	149 (78.8%)	
Female	40 (21.1%)	40 (21.2%)	
Region			1.00
Western Europe	122 (64.2%)	121 (64.0%)	
Eastern Europe	54 (28.4%)	55 (29.1%)	
South America	2 (1.1%)	2 (1.1%)	
Asia	1 (0.5%)	1 (0.5%)	
Others	11 (5.8%)	10 (5.3%)	
KT/V	1.10 (0.97-1.28)	1.13 (0.99-1.26)	0.57
KT/V categories			0.97
< 1.2	129 (67.9%)	130 (68.8%)	
1.2-1.4	36 (18.9%)	36 (19.0%)	
> 1.4	25 (13.2%)	23 (12.2%)	
Body mass index (kg/m²)	24.8 (22.7-26.7)	24.7 (22.8-27.0)	0.76
Albumin (g/dL)	39 (37-41)	40 (38-41)	0.12
hsCRP (mg/L)	7.3 (2.7-20.1)	6.0 (2.9-15.6)	0.53
Diabetes	35 (18.4%)	40 (21.2%)	0.52
Cardiovascular disease	87 (45.8%)	95 (50.3%)	0.41
Study treatment			0.61
Rosuvastatin	96 (50.5%)	90 (47.6%)	
Placebo	94 (49.5%)	99 (52.4%)	

p-value from Wilcoxon test, Fisher's exact test or chi-square test as appropriate.

Supplemental Table S2: Comparison of matching variables between cases and controls in Phase II

Matching variables	Controls (n=199) Median (Q1-Q3) / n (%)	Cases (n=200) Median (Q1-Q3) / n (%)	p-value
Age (years)	61 (55-71)	67 (59-74)	<0.0001
Sex			0.92
Male	114 (57.3%)	116 (58.0%)	
Female	85 (42.7%)	84 (42.0%)	
Region			0.0010
Western Europe	115 (57.8%)	97 (48.5%)	
Eastern Europe	35 (17.6%)	46 (23.0%)	
South America	18 (9.0%)	19 (9.5%)	
Asia	15 (7.5%)	3 (1.5%)	
Others	16 (8.0%)	35 (17.5%)	
KT/V	1.23 (1.02-1.40)	1.22 (1.05-1.36)	0.57
KT/V categories			0.73
< 1.2	85 (45.0%)	91 (47.6%)	
1.2-1.4	56 (29.6%)	58 (30.4%)	
> 1.4	48 (25.4%)	42 (22.0%)	
Body mass index (kg/m²)	24.2 (21.9-27.7)	24.5 (21.5-28.7)	0.40
Albumin (g/dL)	40 (38-42)	39 (36-41)	0.003
hsCRP (mg/L)	3.9 (1.4-9.0)	5.9 (2.0-20.1)	0.0009
Diabetes	49 (24.6%)	74 (37.0%)	0.009
Cardiovascular disease	66 (33.2%)	103 (51.5%)	0.0003
Study treatment			0.23
Rosuvastatin	94 (47.2%)	107 (53.5%)	
Placebo	105 (52.8%)	93 (46.5%)	

p-value from Wilcoxon test, Fisher's exact test or chi-square test as appropriate.

Supplemental Table S3: Comparison of matching variables and plasma microRNA levels between the two study populations (Phase I and Phase II)

Matching variables	Phase I (n=379) Median (Q1-Q3) / n (%)	Phase II (n=399) Median (Q1-Q3) / n (%)	p-value
Age (years)	67 (60-74)	65 (57-72)	0.005
Sex			<0.0001
Male	299 (78.9%)	230 (57.6%)	
Female	80 (21.1%)	169 (42.4%)	
Region			<0.0001
Western Europe	243 (64.1%)	212 (53.1%)	
Eastern Europe	109 (28.8%)	81 (20.3%)	
South America	4 (1.1%)	37 (9.3%)	
Asia	2 (0.5%)	18 (4.5%)	
Others	21 (5.5%)	51 (12.8%)	
KT/V	1.12 (0.98-1.28)	1.22 (1.04-1.38)	<0.0001
KT/V categories			<0.0001
< 1.2	259 (68.3%)	176 (46.3%)	
1.2-1.4	72 (19.0%)	114 (30.0%)	
> 1.4	48 (12.7%)	90 (23.7%)	
Body mass index (kg/m²)	24.7 (22.7-26.9)	24.3 (21.8-28.0)	0.87
Albumin (g/dL)	39 (37-41)	40 (37-42)	0.70
hsCRP (mg/L)	6.4 (2.9-18.1)	4.7 (1.7-15.0)	0.0004
Diabetes	75 (19.8%)	123 (30.8%)	0.0005
Cardiovascular disease	182 (48.0%)	169 (42.4%)	0.005
Study treatment			0.72
Rosuvastatin	186 (49.1%)	201 (50.4%)	
Placebo	193 (50.9%)	198 (49.6%)	
**microNAs**			
miR-186-5p	4.52 (4.04-4.94)	4.39 (3.99-4.78)	0.011
miR-210-3p	4.29 (4.00-4.70)	4.06 (3.77-4.34)	<0.0001
miR-632	4.06 (3.58-4.52)	3.84 (3.39-4.17)	<0.0001

p-value from Wilcoxon test, Fisher's exact test or chi-square test as appropriate.
